# *Nigella sativa* L. (Black Cumin): A Promising Natural Remedy for Wide Range of Illnesses

**DOI:** 10.1155/2019/1528635

**Published:** 2019-05-12

**Authors:** Ebrahim M. Yimer, Kald Beshir Tuem, Aman Karim, Najeeb Ur-Rehman, Farooq Anwar

**Affiliations:** ^1^Department of Pharmacology and Toxicology, College of Health Sciences, Mekelle University, Ethiopia; ^2^Department of Pharmacognosy, College of Health Sciences, Mekelle University, Ethiopia; ^3^Department of Pharmacology, College of Pharmacy, Prince Sattam Bin Abdulaziz University, Al-Kharj, Saudi Arabia; ^4^Department of Chemistry, University of Sargodha, Sargodha, Pakistan

## Abstract

The seed of* Nigella sativa* (*N. sativa*) has been used in different civilization around the world for centuries to treat various animal and human ailments. So far, numerous studies demonstrated the seed of* Nigella sativa *and its main active constituent, thymoquinone, to be medicinally very effective against various illnesses including different chronic illness: neurological and mental illness, cardiovascular disorders, cancer, diabetes, inflammatory conditions, and infertility as well as various infectious diseases due to bacterial, fungal, parasitic, and viral infections. In spite of limited studies conducted so far, the promising efficacy of* N. sativa* against HIV/AIDS can be explored as an alternative option for the treatment of this pandemic disease after substantiating its full therapeutic efficacy. Moreover, the strong antioxidant property of this valued seed has recently gained increasing attention with regard to its potential role as dietary supplement with minimal side effects. Besides, when combined with different conventional chemotherapeutic agents, it synergizes their effects resulting in reducing the dosage of concomitantly used drugs with optimized efficacy and least and/or no toxicity. A number of pharmaceutical and biological properties have been ascribed to seeds of* N. sativa*. The present review focuses on the profile of high-value components along with traditional medicinal and biological principles of* N. sativa *seed and its oil so as to explore functional food and nutraceutical potential of this valued herb.

## 1. Introduction

Plants have long been used as a basis of traditional remedies in the history of mankind and also act as sources of modern medicines. According to the World Health Organization (WHO), more than three-fourths of the communities in resource-limited countries rely upon medicinal plants for their primary health care needs because more than 60% of the societies are unable to have access and/or afford allopathic medicines [1, 2]. In line with the new progress in the area of optimum nutrition, nowadays there is a resurgence of interest in the use of plants as a source of food and medicine [3, 4]. Recently, the usage of phytomedicine has been amplified dramatically for numerous ailments because of not only their easy accessibility and low cost but also the belief that natural remedies have fewer harmful effects as compared to synthetic medicines [5].

The development of new products from natural sources is also encouraged because it is estimated that, of the 300,000 herbal species that exist globally, only 15% have been explored for their pharmacological potential [6]. Among several medicinal plants,* Nigella sativa *L. (Ranunculaceae) has been considered one of the most treasured nutrient-rich herb in history around the world and numerous scientific studies are in progress to validate the traditionally claimed uses of small seed of this species [7, 8].

The maximal nutritional value of black cumin can be linked to the presence of substantial amount of vegetable protein, fiber and minerals, and vitamins. The nutritional composition reported from different sources revealed 20-85% of protein, 38.20% of fat, 7-94% of fiber, and 31.94% of total carbohydrates. Among various amino acids identified, glutamate, arginine, and aspartate while cysteine and methionine were the major and minor amino acids, respectively. Black cumin seeds also contain significant levels of iron, copper, zinc, phosphorus, calcium, thiamin, niacin, pyridoxine, and folic acid [7, 8]. In addition, phytochemical analyses of* N. sativa* displayed the presence of over hundreds of phytoconstituents which include mainly alkaloids, saponins, sterols, and essential oil but the composition of many of these have not been chemically recognized nor have been biologically verified. The* N. sativa* seed contain 26-34% fixed oil of which the major fatty acids are linoleic acid (64.6%) and palmitic acid (20.4%). The seed oil is comprised of 0.4%–2.5% essential oil [9, 10]. Amongst different active constituents reported so far, thymoquinone found as major component of the essential oil is the most bioactive compound and exhibits wide ranging therapeutic benefits [11].

## 2. High-Value Bioactive Compounds (Phytochemicals) in the Seed of* Nigella sativa*

Several bioactive compounds from the seed of* N. sativa* have been reported in the literature; among those the most important bioactive ones are thymoquinones. Other main phytochemicals reported from different varieties of* N. sativa* include sterols and saponins, phenolic compounds, alkaloids, novel lipid constituents and fatty acids, and volatile oils of varying composition [12]. The essential oil composition (0.4-0.45%) reported in various studies represented about forty different compounds, amongst the abundantly constituents identified are* tran*s-anethole,* p*-cymene limonene, carvone, *α*-thujene, thymoquinone (TQ), thymohydroquinone (THQ), dithymoquinone, carvacrol, and *β*-Pinene with various concentration [13–15].

The quantity of most important bioactive constituent, thymoquinone, present in the volatile oil isolated by different extraction methods from the seeds of* N. sativa* varied over a wide range: using SC-CO_2_ (1.06, 4.07 mg/g) [16] and by Soxhlet extraction (2940.43 mg/kg) [17] and (8.8 mg/g) oil [18].

The seed oil fatty acid composition (32-40%) has been reported by various authors to contain mainly, linoleic, linolenic, oleic, palmitoleic, palmitic acids together with arachidonic, eicosadienoic, stearic, and myristic acid [15, 16, 19]. A new dienoate and two known monoesters along with novel lipids have been isolated from the unsaponified extract of the seed, namely methylnonadeca-15,17-dienoate, pentyl hexadec-12-enoate, and pentyl pentadec-11-enoate [20].

Phytosterols are important part of human diet and are gaining greater interest due to their nutraceutical and medicinal benefits in lowering low density lipoprotein and total cholesterol level [21]. Phytosterols are also important as characteristic compounds for assessing the quality of vegetable oils and food labeling. The total sterols content of black cumin seed oil as estimated by different researchers was found to be between 18 and 42% of the unsaponified matter. The major sterols identified were *β*-sitosterol, campesterol, stigmasterol, and 5-avenasterol [19, 22]. Tocopherols exhibited attractive scavenging potentials of free radicals which are believed to terminate lipids peroxidation [23]. The total tocopherol contents of black seed oil reported in varied quantities from diverse sources ranged from 9.15 to 27.92 mg/100 g. Among the foremost tocopherols recognized in black cumin seeds, *α*- and *γ*-tocopherol and *β*-tocotrienol are well recognized [19].

Steroidal glycosides of new and known structures have been isolated from* N. sativa* seeds which include 3-*O*-[*β*-D-xylopyranosyl-(1→2)-*α*-L-rhamnopyranosyl-(1→2)-*β*-D-glucopyranosyl]-11-methoxy-16, 23-dihydroxy-28-methylolean-12-enoate, stigma-5,22-dien-3-*β*-D-glucopyranoside [24], and 3-*O*-[*β*-D-xylopyranosyl-(1→3)-*α*-L-rhamnopyranosyl-(1→4)-*β*-D-glucopy-ranosyl]-11-methoxy-16-hydroxy-17-acetoxy hederagenin [25]. Moreover, alkaloids of diverse types have been isolated from the seeds of black cumin, which include novel Dolabellane-type diterpene alkaloids: nigellamines A_1_, A_2_, B_1_, and B_2_ and nigellamines A3, A4, A5, and C [26, 27] possessing lipid metabolizing property, and indazole class of alkaloids: nigellidine, nigellicine [28, 29], and nigellidine-4-O-sulfite [30].

## 3. Traditional Uses of* Nigella sativa* in Folk Remedies

*Nigella sativa* has been widely used as a spice and flavoring agent in variety of food preparations such as in bread, yogurt, pickles, sauces, and salads. Black seed or black cumin (English),* Habbatul Barakah* (Arabic),* Tikur azmud* (Amharic), has long been used in traditional remedy in the Arabian countries, Far East Asia, Europe, and Africa [31].* Nigella sativa *has also been described as the miraculous plant and considered by earliest herbal specialists as “The herb from heaven” [32]. The Prophet Mohammed (PBUH) had described the curative powers of the black seed as “Hold on to use this black seed, as it has a remedy for every illness except death” [33]. Avicenna, a well-known physician of 10th century famous for his book “The Canon of Medicine,” has recommended use of* Nigella* seeds for enhancement of body's energy and also support during recovery from fatigue and dispiritedness.* Nigella sativa* is also mentioned for its curative property in the Holy Bible and is also labelled as* Melanthion* by Hippocrates and Dioscorides [34, 35].

The medicinal use of black cumin seeds in various traditional herbal systems is known for a wide range of ailments which include different airway disorders, for pain such as chronic headache and back pain, diabetes, paralysis, infection, inflammation, hypertension, and digestive tract related problems administered in different kind of preparations. It has also been used topically where it is applied directly to the blisters, nasal abscesses, orchitis, eczema, and swollen joints [33].

Keeping in view of the numerous traditional medicinal uses of* N. sativa* seeds and its active component, thymoquinone, this valuable herb can be explored as an effective folk medicine with multiple pharmacological actions.

## 4. Pharmacological Activities of* Nigella sativa*

*Nigella sativa* has been broadly studied in the last few decades and studies have reported that it possesses a number of medicinal properties and pharmacological actions. In order to retrieve the relevant literatures with respective subtopics, we have used PubMed, Science Direct, Scopus Google Scholar, and grey literatures using different searching terms such as “*Nigella sativa*” or “Black cumin” or “Black seed” and respective disease conditions. In the case of PubMed searching, we have used the respective “Mesh” terms and text words “tw” in order to retrieve all the relevant articles regardless of time boundaries.

### 4.1. Antioxidant Activity

Oxidative stress and an intensification in the levels of free radicals are amongst the foremost central markers associated with several progressive pathological conditions, including neurological disorder, cancer, aging, and endocrine illness [36]. To date, there has been a growing importance in the therapeutic option of medicinal plants as natural antioxidants. Among the various naturally occurring medicinal plants,* N. sativa* has been reported for its effective antioxidant activities of* in- vivo* and* in- vitro* studies [37].

The concomitant usage of* Allium sativum* and* N. sativa* seed in thirty postmenopausal women after two months of consumption revealed a significant reduction in plasma malondialdehyde (MDA) levels with increased activity in erythrocyte glutathione peroxidase (GSH-Px) and superoxide dismutase [38]. Likewise, the fixed and essential oil of black cumin seed revealed a significant increment of Glutathione-S-transferase (GST), glutathione reductase and GSH-Px against oxidative stress brought by potassium bromate in rats' model [39].

The separate administration of* N. sativa* and nanosized clinoptilolite to Wistar rats also showed significant improvement on antioxidant parameters than concomitant uses of both extracts and diabetic groups [40]. A randomized controlled clinical trial in fifty volunteer obese subjects also demonstrated that* N. sativa* seed oil along with a less caloric diet significantly diminished the superoxide dismutase (SOD) level and body weight as compared to the placebo group in eight weeks' trial [41]. Moreover, the methanolic extract and essential oil fractioned from* N. sativa* seed in atherogenic suspension nourished rats has been reported effectively replenished the plasma total antioxidant power by eighty-eight percent against free radicals [42]. Similarly, the oil of* N. sativa *and thymoquinone administration markedly ameliorated cisplatin-induced alteration on carbohydrate biotransformation and enzymatic and nonenzymatic antioxidant defense system in the gastric mucosa [43]. Hence, the marked antioxidant activity of* N. sativa* and thymoquinone might be a potential newer antioxidant agent and used as essential nutrients for life for health promotion and diseases prevention.

### 4.2. Antidiabetic Activity

Even with the advancement in the management of diabetes mellitus, exploration for innovative agents continues since the existing synthetic agents have numerous limitations [44]. The administration of black cumin seed for one month to streptozotocin-induced diabetic rats displayed a significant reduction of fasting plasma glucose, serum MDA, interleukin-6, and immunoglobulin A, G, and M while substantial increment of endogenous antioxidant enzymes; SOD, Glutathione-S-transferase, and catalase expression were noticed. The histology of pancreas in* N. sativa* treated group also revealed an improvement in the pancreatic *β*-cells degeneration, inflammation, and congestion as compared to diabetic control [45]. The combination of administration of* N. sativa* and* Cinnamomum cassia* extracts (NSCCe) to experimentally STZ-induced diabetic rats also showed significantly stabilized serum glucose concentrations, lipid profile, and renal function parameters as compared to the diabetic control. Significant effects were observed in animals that received combined extract and metformin on these parameters. A substantial reversal of the histopathological pancreatic cell injury was also observed in animals receiving the concomitant extracts of NSCCe [46]. The marked antidiabetic activity upon three-month supplementation of* N. sativa *(2 g/day) along with oral antidiabetic agent in type 2 DM patients has also been reported. In this study,* N. sativa *received group showed significant reduction of fasting plasma glucose, hemoglobin A1c, and TBARBs, while marked elevation of the total antioxidant capacity, SOD, and glutathione levels were noted [47].

Furthermore, an experimental randomized controlled trial of 99 diabetes patients received the placebo and two treatment groups received oral black seed oil. Administration of 1.5 and 3 mL/day of black seed oil for 20 days showed meaningful reduction of glycated hemoglobin A1c and random blood sugar levels [48]. The effect of* N. sativa* seed on the glycemic control of patients with type-2 diabetes (DM-2) was also used as an adjunctive treatment added to their oral hypoglycemic agents.* N. sativa* at a dose of two g/day also influenced substantial reductions in fasting plasma glucose and glycated hemoglobin (HbA1c) without major alteration in body weight [49]. The oil of* N. sativa *(NSO) at 2 mL/kg also was showed to reduce fasting plasma glucose and intensification of insulin levels in diabetic rats compared to control. Diabetic rats that received NSO exhibited substantial improvements in lipid profile and expressive increment of pancreatic and hepatic antioxidant enzymes also augmented the histological image and glycogen contents other than improvements of average pancreatic islet extent than the diabetic groups [50].

The different doses of* N. sativa* seed (1, 2, and 3 g/day) in patients with DM-2 were also evaluated. A one g/day administration increased high-density lipoprotein cholesterol (HDL-c) levels after 3 months while two and three g/day of* N. sativa* seed significantly decreased serum levels of total cholesterol (TC) and triglyceride (TG) as well as low-density lipoprotein cholesterol (LDL-c) and increased plasma HDL-c [51]. In reference to modern scholars' devotion to the likely effects of medicinal herbs in diabetic management, a recent meta-analysis of antidiabetic effects of* N. sativa* [44] also exhibited the maintenance of glucose homeostasis and serum lipid profiles in diabetic human subjects [44, 51].

Generally, the possible antidiabetic mechanisms of* N. sativa *might be mediated via modulation of oxidative status (either through upregulation of endogenous antioxidants or reduction of oxidative species) [45, 47], attenuation of inflammation [45], improvement of lipid profiles, increased good cholesterol (HDL-c), while reducing bad cholesterols (LDL-c, TC, and TG) and body weight [44, 46, 51].

### 4.3. Antihypertensive Activity

Numerous antihypertensive agents have been clinically used to control hypertension and to relieve associated comorbid conditions. However, the effectiveness of these agents is only in 40-60% of hypertensive patients and commonly combination of two or more blood lowering agents from diverse antihypertensive classes is required to attain the desired outcomes [52]. This eventually increases the likelihoods of untoward effects and also raises the cost of therapy. A number of herbal products such as the seed of* N. sativa* have been used and claimed to have positive effects against elevated blood pressure (BP).

According to a nonrandomized controlled trials, 57 patients who were allocated to receive 2 g daily supplementations of black cumin for one year displayed a noticeable reduction in systolic, diastolic, and mean arterial BP, heart rate, TC, LDL-c, the fractions of TC/HDL-c, and LDL-c/HDL-c while serum HDL-c was suggestively raised compared with the corresponding baseline values and the control group [53]. Although a trend towards reduction in BP was observed after* N. sativa *administration, one randomized controlled clinical trial failed to show a significant reduction of BP in elderly patients with hypertension [54]. This might be because of the sample size, dosage (300 mg BID for 4 weeks) of the* N. sativa* used in this study, the severity of hypertension, and study population used. For instance, previous clinical studies conducted by Dehkordi et al. [55] and Qidwai et al. [56] conducted on mild hypertensive patients with the dosage of 200 mg BID for 4 weeks and 500 mg BID for 6 weeks, respectively, showed a significant reduction of SPB.

In addition, it has been employed to determine the blood pressure lowering potential and possible mechanisms of* N. sativa* in rats' model, and it was found that the seed oil and nicardipine received groups' revealed substantial reduction in BP. The BP diminishing effect was related with a reduction in cardiac lipid peroxidation product and inhibitory activity of angiotensin converting enzyme in both groups but plasma nitric oxide level significantly increased in* N. sativa* oil received group than the placebo and nicardipine received groups [57]. Black cumin and its active component, thymoquinone, exhibited a reduction in oxidative stress via calcium channel blockade and increasing urine output activity which might have been linked to reduction in blood pressure [58]. Based on majority of these reports, various preparation of* N. sativa *showed a sustainable reduction of the BP in animal models and clinical studies hence can be explored as a promising basis of natural antihypertensive drugs.

### 4.4. Neuroprotective Effects

Neurological disorder such as depression is amongst the most prevailing illnesses globally. It is principally affected by the hypoactivity of neurotransmitters, particularly owing to inadequate activity of serotonin [59]. Stress is the chief triggering aspect in the initiation of depression and this premise is steadily supported by various clinical observations. Studies in experimental animals displayed that overwhelming stress conditions produce neurochemical modifications and behavioral deficits [60]. A large number of medicinal herbs and their isolated compounds have been revealed to have medicinal benefits and therapeutic potential. Among the promising medicinal plants, black cumin is a worthwhile herb with a rich historical and religious basis to manage depression and many other neurological disorders.

The intragastric supplementation of TQ (20 mg/mL) in aluminum trichloride and D-galactose induced neurotoxicity in rats showed a meaningful improvement of cognition, SOD, and total antioxidant capacity while reducing acetylcholinesterase activities. It also exhibited a reduction in MDA, nitric oxide levels, and tumor necrosis factor-*α* immunoreactivity and amplified brain derived neurotrophic factor and Bcl-2 levels [61]. While the effects of repeated administration of* N. sativa* in rats indicated that, there was an improvement in learning and recall status [62]. In addition, flavonoids isolated from black cumin have been shown to modulate critical neuronal signaling paths involved in the processes of memory and are likely to affect synaptic plasticity and long-standing potentiating mechanisms [63]. The neuropharmacological effects of the seed and oils of* N. sativa* and its active component, TQ, are described in Table 1. Based on the wide ranging neuropharmacological effects, black cumin seed, its oil, and the active principle thymoquinone (TQ) can be explored as a promising natural remedy for improvement of numerous neurological disorders.

### 4.5. Anti-Inflammatory and Analgesic Effects

Inflammation has a key role in various medical conditions such as cystic fibrosis, rheumatoid arthritis, osteoarthritis, asthma, allergies, and cancer which all are associated with acute and/or chronic pain. The existing anti-inflammatory agents commonly comprise classes of drugs that produce severe adverse effects such as gastric ulcer, bone marrow depression, water, and salt retention, resulting from the extended use [80]. Medicinal herbs including black cumin might be a potential source of novel biological compounds that are safer and with fewer side effects. The volatile oil of black cumin and thymoquinone at various doses revealed a dose-reliant anti-inflammatory activity against carrageenan-induced hind paw edema in rats' parallel to indomethacin [81]. The volatile oil of* N. sativa *seed also displayed a substantial pain-relieving effect in acetic acid-induced writhing, formalin, and tail flick tests [82]. As stated by Al-Ghamdi, the water extract of black cumin also retained anti-inflammatory effects in carrageenan-induced paw edema comparable to acetyl salicylic acid at corresponding doses but failed to display antipyretic activity against yeast-induced pyrexia [83]. Furthermore, the alcoholic extract of black cumin exhibited a noteworthy pain-relieving effect in mice as compared to diclofenac sodium [84]. Additional study also showed that essential oil of black cumin has notable activity as a painkiller in acetic acid-induced writing, formalin, and tail flick tests. It was also revealed that this extract might elevate a significant swimming and anoxia tolerance time [85]. The anti-inflammatory action of TQ might be related to inhibition of the oxidative product of arachidonic acid formation, such as thromboxane B2 and leukotriene by blocking both cyclooxygenase and lipoxygenase enzymes [86, 87].

In addition, the action of black cumin seed on tracheal sensitivity and pulmonary inflammation of guinea pigs, which were exposed to breathe Sulphur mustard together with black cumin, displayed expressively lower magnitude compared to that of only Sulphur mustard exposed group [88]. The bronchial relaxation effects of the boiled extract of* N. sativa* in contrast with theophylline were assessed in asthmatic patients and it was found that black cumin extract caused substantial rises in entirely measured respiratory function tests and the starting time of bronchodilator action of the extract was comparable to that of theophylline [89]. The various extracts, oil, and active constituent (*α*-hederin) of* N. sativa *also showed an improvement of tracheal responsiveness and significant anti-inflammatory activity via decreasing the release of histamine and leukotrienes while increasing the PGE2 from the mast cells and perfused lungs in anima model of allergic asthma [90–93]. This antiasthmatic effect is further substantiated by different clinical studies, and majority of them reported that different* N. sativa *preparations showed an improvements of clinical symptoms and pulmonary function as well as various asthma biomarkers [89, 94–97]. These preclinical and clinical studies evidenced the potential antiasthmatic effects of* N. sativa *but further investigations are required to assure its efficacy.

The efficacy of black cumin oil in patients with rheumatoid arthritis (RA) was also evaluated and data from 40 female patients diagnosed with RA who took* N. sativa* oil capsules (500 mg) twice daily exhibited improvement in disease activity score compared to placebo (P < 0.05). Correspondingly, a noticeable improvement was displayed in number of inflamed joints, incidence of morning stiffness, and disease activity after the consumption of black cumin [98].

Chronic inflammation has been implicated in various chronic illnesses [(cancer, cardiovascular disorders, diabetes, Alzheimer's disease, epilepsy, amyotrophic lateral sclerosis, rheumatoid arthritis, and asthma) that involve progressive and irreversible damage to the cell and/or neurons] as well as in many infectious conditions [99, 100]. Therefore, the crucial role of anti-inflammatory actions of different* N. sativa* preparations and TQ might be the possible sources for the development of a new generation of anti-inflammatory agent to treat these wide ranging conditions.

### 4.6. Antimicrobial Activity

Antimicrobials have been the bases of clinical medicine since the second half of the 20th century and have saved prominent number of people from serious microbial infections. Nevertheless, in the late 20th century and the earliest 21st century it has perceived the advent and widespread of antimicrobial resistance in pathogenic microorganisms throughout the globe [101, 102]. The ever-increasing terrorization of microbial infections and antimicrobial resistant bacteria demands for a global struggle to discover for novel solutions that might be grounded on the natural products such as plants, which are selected on the basis of renowned ethnomedicinal use [103, 104]. Among the inspiring medicinal plants, black cumin is the one that displayed strong antibacterial, antifungal, antiviral, and antiparasitic actions.

#### 4.6.1. Antibacterial Activity

Thymoquinone obtained from seeds of* N. sativa* revealed broader spectrum activities against multiple strains of gram-positive and gram-negative bacteria, including* Bacillus*,* Listeria*,* Enterococcus*,* Micrococcus*,* Staphylococcus*,* Pseudomonas*,* Escherichia, Salmonella*,* Serovar*, and* Vibrio parahaemolyticus *in addition to inhibiting bacterial biofilm formation [105]. The methyl alcoholic extract of the seed also displayed a larger inhibition zone on gram-positive (*S. pyogenes*) as compared to gram-negative bacteria (*P. aeruginosa, K. pneumoniae, *and* P. vulgaris*) [106]. For different isolates of methicillin-resistant* S. aureus*, various concentrations of (100%, 80%, 50%, 40%, 30%, and 20%)* N. sativa* oils displayed an expressively higher zone of inhibitions against all the tested bacterial strains [107]. Thymoquinone also revealed a significant bactericidal activity against gram-positive cocci with MICs ranging from 8 to 32 *μ*g/mL and proved the minimum biofilm inhibition concentration at 22 and 60 *μ*g/mL for* S. aureus* and* S. epidermidis,* respectively [108]. Moreover, black seed (2 g/day) owed clinically valuable anti*-H. pylori* effect comparable to triple therapy [109] and this can provide a scientific basis for the exploration of potential uses of this valued seed for the treatment of* H. pylori*-induced gastric ulcers.

#### 4.6.2. Antifungal Activity

The essential oil of* N. sativa* of different origins has been reported to possess moderate inhibitory action against pathogenic strains of yeasts, dermatophytes and nondermatophytic filamentous fungi along with aflatoxin-producing fungi. The* N. sativa* treatment targeted the cell wall, plasma membrane, and membranous organelles, mainly in the nuclei and mitochondria as were evident in the morphology of these toxigenic fungi [110]. Moreover, different extracts of black cumin and TQ exhibited powerful fungicidal activity against dermatophyte strains including* Trichophyton mentagrophytes* and* Microsporum gypseum* superior to fluconazole, but lesser than that of ketoconazole [111]. Thymoquinone also arrested the growth of* Aspergillus niger* and* Fusarium solani* comparable to Amphotericin-B [112] and was effective against* C. albicans*,* C. tropicalis*, and* C. krusei* [113]. Similarly, as stated by Taha et al., the active constituent of black cumin such as TQ, thymohydroquinone, and thymol revealed potent antifungal effect against several clinically isolated fungal strains including dermatophytes, molds, and yeasts [114]. As a potential candidate with multiple antimicrobial activities,* N. sativa* can also be explored as a natural preservative and food additive to protect foods from spoilage.

#### 4.6.3. Antiviral Activity

*N. sativa *seed oil was found to suppress viral load in murine model: cytomegalovirus infected mice to undetectable level in the liver and spleen in 10 days' intraperitoneal administration. This was possibly due to the increase in number and function of CD4^+ve^ T cells and increased production of interferon- (INF-) gamma [115]. Interestingly, patients (30) with hepatitis C virus (HCV) infection, who were not eligible for IFN-*α*/ribavirin therapy showed significant improvement in HCV viral load (16.67% became seronegative and 50% showing significant decrement) and proved laboratory parameter like total protein, red blood cell, and platelet count, decreased fasting blood glucose, and postprandial glucose in both diabetic and nondiabetic HCV patients and reduced lower-limb edema after they are managed with black cumin seed oil [116].

According to a case report conducted by Onifade et al., after treatment with 10 mL of black seed twice daily for 6 months, a complete regaining and seroreversion of a 46-year-old HIV positive patient was evidenced [117]. In addition, a 27-year-old HIV infected woman was diagnosed during ante-natal care; she was not eligible for antiretroviral therapy; hence herbal therapist initiated her on black cumin and honey mixture (10 mL) thrice daily for a year. The repeat serology assessments for HIV infection became negative with undetectable viral load. The woman also got 3 children (2007, 2010, and 2012) that all were breastfed and none of the children infected with HIV and her repeat CD4 count was not less than 750 cells/*μ*L [118]. Nowadays HIV/AIDS is a serious global threat and in this regard,* N. sativa* can be a promising natural therapy to cure such a chronic infectious disease, after validating its full therapeutic efficacy by further investigations.

#### 4.6.4. Antiparasitic Activity

*Nigella sativa *seeds have shown schistosomicidal properties against* Schistosoma mansoni* (*in vitro*), through a strong biocidal effect against all stages of the parasite and an inhibitory effect on egg-laying of adult female worms [119, 120]. An ointment of* N. sativa *seed significantly contracted and inhibited the inflammatory reactions to cutaneous leishmaniasis produced experimentally in mice by a subcutaneous inoculation of* Leishmania major* at the abaxial base of the tail [121].* N. sativa* extract at a dose of 1.25 g/kg prominently lowered* Plasmodium yoelii* infection in mice by 94%; however, the effect of chloroquine was only 86% as compared to the untreated group. In addition, methanolic extract of* N. sativa *revealed higher parasite clearance and restoration of altered biochemical indicators by* P. yoelii* infection than chloroquine [122]. Thus, considering* N. sativa *for future antiparasitic agents will have a very important input after conduction of further investigation of its curative, prophylactic and chemopreventive activity particularly in the era of emerging antimalarial drug resistance.

### 4.7. Anticancer Activity

Cancer is a bigger challenge in medical science as the incidence of this health disorder is rapidly growing across the world. This prompts the efforts to search some effective natural anticancer therapies alternative to currently employed chemotherapies with limited applications. The effect of black seed in different types of cancer cells is summarized in Table 2. As there are ten cancer hallmarks which are common to most tumors, TQ, a major active component of* N. sativa*, plays great role in affecting all markers of cancer [123].

### 4.8. Effects on Male Infertility

Infertility is the incapability of a copulate to attain offspring after 12 months of intercourse without contraception. It is more prevalent among men than women [142]. Sperm dysfunction is the main problem related with men infertility which accounts 60% of all reasons. The structure, function, motility, and survival of sperm are deleteriously affected by oxidative stress that prominently leads to infertility. Hence, increasing spermatozoa counts, functionality, and sperm quality using antioxidants can improve fertility status [143, 144]. Evidence proves that some herbal medicines can reduce negative effects of oxidative stress by salvaging free radicals [145]. Among the various traditional plants,* N. sativa* was found to exhibit remarkable antioxidant effect [146].

Alcoholic extract of* N. sativa* indicated remarkable increment in the production of viable and motile sperm cells, enhanced epididymal sperm reservation, weight gaining of reproductive organs, blood testosterone density, gonadotropins content, amount of mature Leydig cells, and fertility indexes compared to the control group in male rats [147]. According to Mohammad et al., black cumin thought to trigger a rise in spermatogenesis hormones on pituitary gland, and an increase in the weight of reproductive organs. The study also reveals that* N. sativa* can affect oxidative phosphorylation enzymes and increase sperm motility [147]. In addition, a randomized, double-blind, placebo-controlled clinical trial was conducted on 68 Iranian infertile men and half of them receive 2.5 mL of black seed oil and the remaining received placebo twice daily for two months. The amount and the motility of sperm and the content of semen volume were raised significantly in black seed oil treated group compared with placebo group after two months of therapy [148]. This indicates that* N. sativa* can be a potential source for development of natural* aphrodisiac *agents.

## 5. Toxicological Properties

The acute oral toxicity of active constituents of black cumin seed, TQ, lethal dose 50 (LD_50_) value has been reported to be 2.4 g per kg of body weight of Swiss albino mice, whereas the instant behavioral alteration at two and three g per kg of body weight of the composite was hypoactivity and trouble in breathing, while late toxicities comprising a substantial lessening in the virtual organ weight and glutathione distribution of the hepatic, renal, and cardiovascular system have been reported [149]. Daily administration of aqueous extract (AqE) of* N. sativa* to mice for six weeks led to death of one mouse after 2 weeks of treatment with 6.4 g/kg of AqE. On the other hand, 2 and 3 mice experienced death at 3rd and 5th weeks while they received 21 g/kg and 60 g/kg of the extract, respectively. Otherwise, no other deaths were recorded for the application of other doses used [150]. In addition, the subchronic toxicity study in mice treated with 30, 60, and 90 mg/kg/day of TQ for 90 days resulted in no mortality or signs of toxicity but substantial decrement of fasting plasma glucose and also showed no change in toxicological significance in body organs and histological investigation [149]. The toxicity of the fixed oil of black cumin in mice and rats was also examined and the LD_50_ values were found to be 28.8 ml/kg and 2.06 ml/kg when given by oral and intraperitoneal routes, respectively. Chronic toxicity was also studied in rats treated daily with an oral dose of 2 ml/kg for 12 weeks' black cumin oil, while alterations in vital liver enzyme levels and histopathological modifications (heart, liver, kidneys, and pancreas) were not detected [151]. The minor and/or negligible toxicological effects and wider therapeutic margin of* N. sativa* and its active constituents, thymoquinone, as evident by various scientific studies support its safe use for the long-term traditional food and medicinal purposes.

## 6. Conclusion and Future Prospects

Traditional medicinal plants have received much attention due to several factors such as low cost, ease of access, and lower adverse effect profiles as compared to synthetic medicines. Besides, various medicinal floras and their products are used on the basis of religious and cultural traditions. Among various plants, black cumin has been used by diverse human cultures around the world especially in Muslim population for centuries to treat numerous ailments. To date, a number of studies showed that black seed and its component including TQ have revealed a remarkable natural therapy for treatment of a wide range of illnesses including chronic noninfectious (neurologic disorders, DM, hypertension, dyslipidemia, inflammatory disorders, cancer, etc.) and infectious disease (bacterial, fungal, viral, and parasitic infections). Both animal and human studies also showed that black seed and TQ have potential to treat male infertility and their antioxidant activities have recently gained greater attention due to their role as dietary supplements with minimal side effects. Furthermore, when combined with different conventional chemotherapeutic agents, they synergize the effects which may reduce the dosage of the concomitantly used medicines and optimizing efficacy versus toxicity and it might also overcome drug resistance problem. Therefore, having wider safety margins and praiseworthy efficacy against wide range of maladies, it would be a potential herbal remedy to be assessed under clinical trial for numerous conditions. Isolation of novel bioactive components from black cumin and its oil and studies of their therapeutic effects using specific clinical models are further recommended.

## Figures and Tables

**Table 1:** The effects of *N. sativa* and its active component, thymoquinone (TQ) on neurological and mental disorders.

Neurological or mental Disorders	Model used and intervention (s)	Finding (mechanism)	References
Alzheimer's disease (AD)	Lipopolysaccharide-induced AD in mice, received TQ (2.5 & 5mg/kg) for 7 days.	(i) ↓ TBARS & 5-LOX levels (ii) ↑ GSH extent and SOD action (iii) Causes disaggregation of A*β* peptide (iv) prevents declining of neurons (v) Slows degeneration of cognitive ability	[64, 65]
	A*β*-induced neurotoxicity (analyzed by culturing hippocampus and cortical neurons). TQ is administered along with A*β*_1−42_ for 72 hours	(i) Reducing A*β*-induced neurotoxicity. (Improved cell viability) by: (ii) Inhibiting mitochondrial membrane potential depolarization (iii) Hindering reactive oxygen species generation	[66]

Parkinson's disease (PD)	1-methyl-4-phenylpyridinium (MPP^+^) and rotenone-induced neurotoxicity in PD model, cultures were treated with TQ (0.01, 0.1, 1 and, 10 *μ*M) on day 8th for 4 days.	(i) Rescued dopaminergic neurons through: (ii) Its antioxidant and anti-inflammatory effects	[67]
	Experimental model of early PD induced by 6-hydroxydopamine neurotoxicity, pretreatment of daily TQ (5 & 10 mg/kg) and one additional dose after surgery were used.	(i) ↓ MDA level (ii) Prevents loss of neurons in substantia nigra (iii) Protects hippocampal & human induced pluripotent stem cell against *α*-synuclein induced synaptic toxicity	[68, 69]

Depression and anxiety	(i) Open field and elevated plus maze models; forced swim test (ii) Locomotor behavior in familiar and new environment in rats, *N. sativa * oil (0.1 mL/day) aqueous seed extract (2 mL/day) orally for 4-6 weeks	(i) ↑ in open field activity & struggling time (ii) ↑ 5-HT (iii) ↓ 5HIAA level in the brain (iv) ↑ tryptophan level in plasma & brain (v) ↑ locomotors activity in novel environment (vi) ↑ brain DA level	[59, 70, 71]
	Stressed and unstressed mice, 10 and 20 mg/kg of TQ for 4 weeks	Unstressed mice: at 10 & 20 mg/Kg showed anti-anxiety (i) without altering nitrite levels (ii) ↑ GABA content (only 20mg/Kg). Stressed mice: 20 mg/kg showed anxiolytic effects with (i) ↓ plasma nitrite level (ii) Reversal of reduced GABA	[72]
	Randomized control trial on healthy human subjects, *N. sativa* capsule (500 mg) daily for 4 weeks.	(i) Stabilize disturbed mood (ii) ↓ anxiety (iii) Modulate memory positively	[73]

Epilepsy	Pentylenetetrazole-induced seizure, *N. sativa* oil; TQ	(i) Prevented seizure occurrence (ii) ↓ Reactive oxygen species generation (iii) Reduced seizure score (iv) Showed additive effects with phenobarbitone	[74–76]
	Double-blinded placebo randomized control trial (refractory epilepsy), TQ as adjunctive therapy for 4 weeks	(i) Significant reduction of seizure frequency (those who received combination therapy)	[77]

Opioid dependence and Tolerance	Morphine brought tolerance and dependency in mice, 4mL/kg of *N. sativa* oil along with morphine (5mg/kg)	(i) Attenuated the development of tolerance (ii) Inhibited nitric oxide overproduction (iii) ↓ in brain MDA level	[78]
	Randomized trial (on 35 known addicts of opiates), 500 mg *N. Sativa *three times daily	(i) ↓ the withdrawal effects significantly (ii) ↑ appetite (no significant weight gain) (iii) No changes in physiological parameters (blood pressure, pulse and respiratory rate)	[79]

TBARs= Thiobarbituric acid reactive substances, GABA= gamma amino butyric acid, 5-HT= 5 hydroxytryptamine, MDA= malondialdehyde, DA= dopamine, 5HIAA= 5 hydroxyindoleacetic acid, GSH= glutathione peroxidase, SOD= superoxide dismutase, TQ= thymoquinone, A*β*= beta amyloid peptides, ↑=increase, ↓=decrease.

**Table 2:** Effects of *N. sativa* and thymoquinone against various types of cancer models and their effects on anticancer agents.

Cancer models or effects of anticancer agents		Intervention (s)	Findings (Mechanisms)	References
*In vitro *studies	Doxorubicin-resistant human breast cancer cells line (MCF-7/DOX cells)	TQ (25, 50 or 100 *µ*M) for 48 hours & NSO Nano emulsion	(i) Concentration dependent growth inhibition (ii) Induce apoptosis, p53 protein (iii) Upregulation of PTEN (inhibit PI3K/Akt pathway)	[124, 125]
	Human cervical squamous cancer cells	TQ (1.0 to 30 *μ*g/mL) for 24, 48 and 72 hours	(i) More cytotoxic than cisplatin towards this cancerous cell (but less cytotoxicity towards normal cells) (ii) Downregulates Bcl-2 protein	[126]
	Myeloblastic leukemia (HL-60 cells)	TQ	(i) Induces apoptosis, disrupts mitochondrial membrane potential, triggers the activation of caspases 3, 8 & 9 in HL-60 cells	[127]
	Human bladder cancer cells (T24 and 253J)	TQ (20-160 *µ*mol/L) for different periods (24h, 48h, and 72h)	(i) TQ showed marked cytotoxicity on bladder cancer cells (ii) It inhibited cancerous cells rapid multiplication and evoked apoptosis via activation of caspase. (iii) TQ also resulted in activation of ER stress, mitochondrial disturbance and enhanced mitochondrial mediated apoptotic path.	[128]
	Renal cell cancer (RCC) cell lines (786-O and ACHN)	TQ (40 *μ*mol/L) for 24 hours	(i) TQ suppressed migration, invasion and epithelial-mesenchymal transition in RCC cells. (ii) TQ exhibited significant inhibition of the metastasis of RCC cells through induction of autophagy via AMPK/mTOR signalling.	[129]
	Human renal tubular epithelial cell (HK2) and the human RCC cell lines (769-P & 786-O)	TQ (0.5, 1, 2.5, 5, 10, 15 & 20 *µ*M) at various durations (0, 24, 48 & 72 h).	(i) TQ markedly inhibited the migration and invasion of the human RCC 769-P and 786-O cell lines. (ii) TQ also increased the expression of E-cadherin and reduced the expression of Snail, ZEB1 and vimentin at the mRNA as well as protein levels in dose-dependent fashion. (iii) As a result, the extents of phosphorylation of hepatic kinase B1 and AMPK were upregulated.	[130]
	Human prostate cancer cell lines (DU145 and C3)	TQ (2.5, 5.0 & 10 *µ*M) for 24 hours.	(i) TQ substantially arrested the proliferation of prostate cancer. (ii) It inhibited the migrating and invading capability of prostate cancer DU145 and PC3 cells. (iii) TQ also downregulated the expression of TGF-*β*, Smad2 and Smad3 in prostate cancer cells.	[131]
	Hepatocellular cancer cell line (HepG2)	TQ (3–24 *µ*M) for 24 hours.	(i) Decreased both the no. of viable HepG2 cells and the levels (ii) TQ induced cell cycle arrest and apoptosis (iii) Increased total antioxidant status (dose dependently) (iv) TQ reduced the release of VEGF of HepG2 cells	[132]

*In vivo *studies	Diethyl nitrosamine inducedhepatocarcinogenesis in Wistar rats	Ethanolic extract of NS (250 mg/kg) for 5 consecutive days.	(i) The chemical induced increment of liver weight, hepato-somatic indices, serum AFP and VEGF levels, and hepatic HGF*β* protein expression were significantly reversed by the extract. (ii) The histopathological alteration of the livers due to the chemical was decreased in NS extract received rats without harmful effects.	[133]
	Orthotopic model inmice [triple-negative breast cancer (TNBC) cell lines]	TQ (20 or 100 mg/k) once every 3 days	(i) TQ markedly reduced the growth of MDA-MB-231 tumor. (ii) TQ decreased TNBC cell viability and proliferation as well as the migration and invasion of TNBC cells. (iii) TQ also downregulated the expression of eEF-2K (via modulation of the NF-*κ*B/miR-603), Src/FAK, and Akt in TNBC cells.	[134]
	Colon carcinogenesis of rats model	NSO for 14 weeks	(i) NSO revealed a significant antiproliferative activity in both initiation and post-initiation phases (ii) Inhibited colon carcinogenesis of rats mainly in the post-initiation stage with no evident of adverse effects	[135]
	Mouse model of colorectal carcinogenesis & C26 cell	TQ (5 mg/kg) for 3 weeks & TQ (0, 20, 40, 60 *μ*M) *in vitro*	(i) TQ reduced tumor multiplicity (ii) TQ impeded tumor growth and induce apoptosis in HCT116 xenografts (iii) Sub-cytotoxic conc. of TQ (40*μ*M) also reduced C26 cell invasion (iv) Anti-neoplastic and pro-apoptotic p53-dependent mechanism	[136]
	Rat multi-organ carcinogenesis	NSO for 30 weeks	(i) Reduction in malignant and benign colon tumor sizes, tumors in the lungs and in diverse parts of the alimentary canal principally the oesophagus and fore stomach	[137]

Effect on anti-cancer drugs	Cyclophosphamide Induced toxicity (abnormal RF & LFT and reduced Hgb) in rat	NSO (1ml/kg) and TQ (10 mg/kg) EOD for 12 days	(i) Substantial reduction in overall cyclophosphamide induced toxicity in both NSO and TQ treated groups.	[138]
	Antitumor Effect of TQ and gemcitabine on xenograft mouse and PANC-1, AsPC-1 and BxPC-3 cell lines of pancreatic cancer models,	TQ (0–50 *µ*mol/L) & 1.0 mg/ mouse daily	(i) TQ pre-treatment synergistically increased the gemcitabine actions of apoptotic and tumor growth inhibition of pancreatic cancer cells. (ii) Concomitant uses resulted in the change of several molecular signaling, including the downregulation of Notch1, NICD associated with up-regulation of PTEN, via the inactivation of Akt/mTOR/S6 signaling. (iii) TQ and gemcitabine also induced suppression of anti-apoptotic Bcl-2, Bcl-xL, XIAP and overexpression and activation Caspase-3, Caspase-9, & Bax.	[139]
	Cytotoxicity assay of TQ and paclitaxel on mouse breast cancer cell line (4T1) and animal models	TQ (6.25, 12.5, 25, 50, & 100 *μ*M) for 24 & 48 hours; (0.64, 2.4 & 3.2 mg/kg of mouse body weight).	(i) TQ induced marked cytotoxicity and apoptosis, while inhibiting wound healing and migration of 4T1 cells. (ii) Co-administration of TQ and paclitaxel significantly induced cytotoxicity and apoptosis compared to separate administration. (iii) The combination of paclitaxel and the lower dose TQ markedly inhibited the tumor growth. (iv) Both agents also modulated the apoptosis genes, p53 and JAK-STAT signaling, while overexpressing the levels of Caspase-3, Caspase-7, and Caspase-12.	[140]
	Anti-tumor activity of TQ and topotecan in colorectal cancer cell line (HT-29)	TQ (40, 55 & 60 *µ*M)	(i) TQ significantly enhanced the anti-tumor effect of non-cytotoxic dose of topotecan. (ii) Both drugs induced apoptosis via a p53-independent mechanism, while the expression of p21 was only noted in TQ therapy. (iii) TQ improved the effectiveness of topotecan by inhibiting proliferationand lowering toxicity via p53- and Bax/Bcl2-independent mechanisms.	[141]

AMPK: Adenosine monophosphate-activated protein kinase, NS: *Nigella sativa*, NSO: *Nigella sativa* oil, TQ: Thymoquinone, PTEN: phosphatase and tensin homolog, MCF-7: Michigan Cancer Foundation-7, EOD: every other day, mTOR: Mammalian Target of Rapamycin.

## Abbreviations

AqE: Aqueous extract
*N. sativa*: * Nigella sativa*
TQ: Thymoquinone
LD: Lethal dose
PTEN: Phosphatase and tensin homolog
MCF-7: Michigan Cancer Foundation-7
TBARs: Thiobarbituric acid
GABA: Gamma amino butyric acid
5-HT: 5-Hydroxytryptamine
MDA: Malondialdehyde
DA: Dopamine
5HIAA: 5 Hydroxyindoleacetic acid
GSH-Px: Glutathione peroxidase
SOD: Superoxide dismutase
A*β*: Beta amyloid peptides
HCV: Hepatitis C virus
INF: Interferon
MIC: Minimum inhibitory concentration
RA: Rheumatoid arthritis
BP: Blood pressure
DM: Diabetic mellitus
CQ: Chloroquine.

## References

[1] World Health Organization (2017). *Traditional Medicine Fact sheet No 134*.

[2] Jamshidi-Kia F., Lorigooini Z., Amini-Khoei H. (2018). Medicinal plants: Past history and future perspective. *Journal of HerbMed Pharmacology*.

[3] Anwar F., Anwar F., Muhammad G. (2016). Capparis spinosa L.: A plant with high potential for development of functional foods and nutraceuticals/pharmaceuticals. *International Journal of Pharmacology*.

[4] Hassanien M. F. R., Assiri A. M. A., Alzohairy A. M., Oraby H. F. (2015). Health-promoting value and food applications of black cumin essential oil: an overview. *Journal of Food Science and Technology*.

[5] Adib-Hajbaghery M., Rafiee S. (2018). Medicinal plants use by elderly people in Kashan, Iran. *Nursing and Midwifery Studies*.

[6] De Luca V., Salim V., Atsumi S. M., Yu F. (2012). Mining the biodiversity of plants: A revolution in the making. *Science*.

[7] Takruri H. R. H., Dameh M. A. F. (1998). Study of the nutritional value of black cumin seeds (Nigella sativa L). *Journal of the Science of Food and Agriculture*.

[8] Ramadan M. F. (2007). Nutritional value, functional properties and nutraceutical applications of black cumin (Nigella sativa L.): an overview. *International Journal of Food Science & Technology*.

[9] Mamun M. A., Absar N. (2018). Major nutritional compositions of black cumin seeds cultivated in Bangladesh and the physicochemical characteristics of its oil. *International Food Research Journal*.

[10] Ghahramanloo K. H., Kamalidehghan B., Akbari Javar H., Teguh Widodo R., Majidzadeh K., Noordin M. I. (2017). Comparative analysis of essential oil composition of iranian and indian nigella sativa L. Extracted using supercritical fluid extraction and solvent extraction. *Drug Design, Development and Therapy*.

[11] Haseena S., Aithal M., Das K. K., Saheb S. H. (2015). Phytochemical analysis of Nigella sativa and its effect on reproductive system. *Journal of Pharmaceutical Sciences and Research*.

[12] Botnick I., Xue W., Bar E. (2012). Distribution of primary and specialized metabolites in *Nigella sativa* seeds, a spice with vast traditional and historical uses. *Molecules*.

[13] Ainane T., Askaoui Z., Elkouali M. (2014). Chemical composition and antibacterial activity of essential oil of Nigella sativa seeds from Beni Mellal (Morocco): What is the most important part, Essential Oil or the rest of seeds?. *Journal of Materials and Environmental Science*.

[14] Benkaci-Ali F., Akloul R., Boukenouche A., Pauw E. D. (2013). Chemical composition of the essential oil of nigella sativa seeds extracted by microwave steam distillation. *Journal of Essential Oil Bearing Plants*.

[15] Isik S., Kartal M., Erdem S. A. (2017). Quantitative analysis of thymoquinone in Nigella Sativa L. (Black Cumin) seeds and commercial seed oils and seed oil capsules from Turkey. *Ankara Üniversitesi Eczacılık Fakültesi Dergisi*.

[16] Solati Z., Baharin B. S., Bagheri H. (2014). Antioxidant property, thymoquinone content and chemical characteristics of different extracts from *Nigella sativa* L. seeds. *Journal of the American Oil Chemists' Society*.

[17] Herlina, Aziz S. A., Kurniawati A., Faridah D. N. (2017). Changes of thymoquinone, thymol, and malondialdehyde content of black cumin (Nigella sativa L.) in response to Indonesia tropical. *Journal of Biosciences*.

[18] Salea R., Widjojokusumo E., Hartanti A. W., Veriansyah B., Tjandrawinata R. R. (2013). Supercritical fluid carbon dioxide extraction of Nigella sativa (black cumin) seeds using taguchi method and full factorial design. *Biochemical Compounds*.

[19] Matthaus B., Özcan M. M. (2011). Fatty acids, tocopherol, and sterol contents of some nigella species seed oil. *Czech Journal of Food Sciences*.

[20] Mehta B. K., Verma M., Gupta M. (2008). Novel lipid constituents identified in seeds of Nigella sativa (Linn). *Journal of the Brazilian Chemical Society*.

[21] San Mauro-Martín I., Blumenfeld-Olivares J. A., Garicano-Vilar E., Cuadrado M. Á., Ciudad-Cabañas M. J., Collado-Yurrita L. (2018). Differences in the effect of plant sterols on lipid metabolism in men and women. *Topics in Clinical Nutrition*.

[22] Cheikh-Rouhou S., Besbes S., Hentati B., Blecker C., Deroanne C., Attia H. (2007). *Nigella sativa* L.: chemical composition and physicochemical characteristics of lipid fraction. *Food Chemistry*.

[23] Zaunschirm M., Pignitter M., Kienesberger J. (2018). Contribution of the ratio of tocopherol homologs to the oxidative stability of commercial vegetable oils. *Molecules*.

[24] Mehta B. K., Mehta P., Gupta M. (2009). A new naturally acetylated triterpene saponin from Nigella sativa. *Carbohydrate Research*.

[25] Mehta B. K., Pandit V., Gupta M. (2009). New principles from seeds of Nigella sativa. *Natural Product Research (Formerly Natural Product Letters)*.

[26] Morikawa T., Xu F., Kashima Y., Matsuda H., Ninomiya K., Yoshikawa M. (2004). Novel dolabellane-type diterpene alkaloids with lipid metabolism promoting activities from the seeds of Nigella sativa. *Organic Letters*.

[27] Morikawa T., Xu F., Ninomiya K., Matsuda H., Yoshikawa M. (2004). Nigellamines A3, A4, A5, and C, new dolabellane-type diterpene alkaloids, with lipid metabolism-promoting activities from the Egyptian medicinal food black cumin. *Chemical & Pharmaceutical Bulletin*.

[28] Atta-ur-Rahman M. S., Cun-Heng H., Clardy J. (1992). Isolation and structure determination of Nigellicine, a novel alkaloid from the seeds of *Nigella sativa*. *Journal of Natural Products*.

[29] Atta-ur-Rahman M. S., Hasan S. S., Choudhary M. I., Ni C.-Z., Clardy J. (1995). Nigellidine - A new indazole alkaloid from the seeds of Nigella sativa. *Tetrahedron Letters*.

[30] Ali Z., Ferreira D., Carvalho P., Avery M. A., Khan I. A. (2008). Nigellidine-4-o-sulfite, the first sulfated indazole-type alkaloid from the seeds of Nigella sativa. *Journal of Natural Products*.

[31] Javed S., Shahid A. A., Haider M. S. (2010). Nutritional, phytochemical potential and pharmacological evaluation of Nigella Sativa (Kalonji) and Trachyspermum Ammi (Ajwain). *Journal of Medicinal Plants Research*.

[32] Ahmad I., Tripathi J., Manik S., Umar L., Rabia J. (2013). Preliminary phytochemical studies of the miracle herb of the century, Nigella sativa L. (black seed). *Indo American Journal of Pharmaceutical Research*.

[33] Bukhari A. A. Sahih-ul-Bukhari. http://quranx.com/Search?q=Black+cumin&context=Hadith.

[34] Nadkarni K., Nadkarni K. M. (1976). Crocus sativus, *Nigella sativa*. *Indian Material Medica*.

[35] Tariq M. (2008). Nigella sativa seeds: folklore treatment in modern day medicine. *Saudi Journal of Gastroenterology*.

[36] Lupoli F., Vannocci T., Longo G., Niccolai N., Pastore A. (2018). The role of oxidative stress in Friedreich's ataxia. *FEBS Letters*.

[37] Ozdemir N., Kantekin-Erdogan M. N., Tat T., Tekin A. (2018). Effect of black cumin oil on the oxidative stability and sensory characteristics of mayonnaise. *Journal of Food Science and Technology*.

[38] Mostafa R. M., Moustafa Y. M., Mirghani Z., AlKusayer G. M., Moustafa K. M. (2013). Antioxidant effect of garlic (*Allium sativum*) and black seeds (*Nigella sativa*) in healthy postmenopausal women. *SAGE Open Medicine*.

[39] Sultan M. T., Butt M. S., Karim R. (2015). Nigella sativa fixed and essential oil modulates glutathione redox enzymes in potassium bromate induced oxidative stress. *BMC Complementary and Alternative Medicine*.

[40] Omidi H., Khorram S., Mesgari M., Asghari-Jafarabadi M., Tarighat-Esfanjani A. (2017). Effects of separate and concurrent supplementation of Nano-sized clinoptilolite and Nigella sativa on oxidative stress, anti-oxidative parameters and body weight in rats with type 2 diabetes. *Biomedicine & Pharmacotherapy*.

[41] Namazi N., Mahdavi R., Alizadeh M., Farajnia S. (2015). Oxidative stress responses to Nigella sativa oil concurrent with a low-calorie diet in obese women: A randomized, double-blind controlled clinical trial. *Phytotherapy Research*.

[42] Ahmad S., Beg Z. H. (2016). Evaluation of therapeutic effect of omega-6 linoleic acid and thymoquinone enriched extracts from Nigella sativa oil in the mitigation of lipidemic oxidative stress in rats. *Nutrition Journal *.

[43] Shahid F., Farooqui Z., Khan A. A., Khan F. (2018). Oral Nigella sativa oil and thymoquinone administration ameliorates the effect of long-term cisplatin treatment on the enzymes of carbohydrate metabolism, brush border membrane, and antioxidant defense in rat intestine. *Naunyn-Schmiedeberg's Archives of Pharmacology*.

[44] Daryabeygi-Khotbehsara R., Golzarand M., Ghaffari M. P., Djafarian K. (2017). Nigella sativa improves glucose homeostasis and serum lipids in type 2 diabetes: A systematic review and meta-analysis. *Complementary Therapies in Medicine*.

[45] El Rabey H. A., Al-Seeni M. N., Bakhashwain A. S. (2017). The antidiabetic activity of *Nigella sativa* and propolis on streptozotocin-induced diabetes and diabetic nephropathy in male rats. *Evidence-Based Complementary and Alternative Medicine*.

[46] Kaur G., Invally M., Khan M. K., Jadhav P. (2018). A nutraceutical combination of Cinnamomum cassia & Nigella sativa for Type 1 diabetes mellitus. *Journal of Ayurveda and Integrative Medicine*.

[47] Kaatabi H., Bamosa A. O., Badar A. (2015). Nigella sativa improves glycemic control and ameliorates oxidative stress in patients with type 2 diabetes mellitus: Placebo controlled participant blinded clinical trial. *PLoS ONE*.

[48] Rachman P. N. R., Akrom, Darmawan E. The efficacy of black cumin seed (*Nigella sativa*) oil and hypoglycemic drug combination to reduce HbA1c level in patients with metabolic syndrome risk.

[49] Bamosa A., Kaatabi H., Badar A. (2015). Nigella sativa: A potential natural protective agent against cardiac dysfunction in patients with type 2 diabetes mellitus. *Journal of Family and Community Medicine (JFCM)*.

[50] Abdelrazek H. M. A., Kilany O. E., Muhammad M. A. A., Tag H. M., Abdelazim A. M. (2018). Black seed and thymoquinone improved insulin secretion, hepatic glycogen storage, and oxidative stress in streptozotocin-induced diabetic male wistar rats. *Oxidative Medicine and Cellular Longevity*.

[51] Kaatabi H., Bamosa A. O., Lebda F. M., Al ELq A. H., Al-Sultan (2012). Favorable impact of Nigella sativa seeds on lipid profile in type 2 diabetic patients. *Journal of Family and Community Medicine*.

[52] Vasant O. K., Vijay B. G., Virbhadrappa S. R., Dilip N. T., Ramahari M. V., Laxamanrao B. S. (2012). Antihypertensive and diuretic effects of the aqueous extract of *Colocasia esculenta* Linn. leaves in experimental paradigms. *Iranian Journal of Pharmaceutical Research*.

[53] Badar A., Kaatabi H., Bamosa A. (2017). Effect of Nigella sativa supplementation over a one-year period on lipid levels, blood pressure and heart rate in type-2 diabetic patients receiving oral hypoglycemic agents: Nonrandomized clinical trial. *Annals of Saudi Medicine*.

[54] Rizka A., Setiati S., Lydia A., Dewiasty E. (2017). Effect of *Nigella sativa* seed extract for hypertension in elderly: a double-blind, randomized controlled trial. *Acta Medica Indonesiana*.

[55] Dehkordi F. R., Kamkhah A. F. (2008). Antihypertensive effect of *Nigella sativa* seed extract in patients with mild hypertension. *Fundamental & Clinical Pharmacology*.

[56] Qidwai W., Hamza H. B., Qureshi R., Gilani A. (2009). Effectiveness, safety, and tolerability of powdered Nigella sativa (kalonji) seed in capsules on serum lipid levels, blood sugar, blood pressure, and body weight in adults: results of a randomized, double-blind controlled trial. *Journal of Alternative and Complementary Medicine*.

[57] Jaarin K., Foong W. D., Yeoh M. H. (2015). Mechanisms of the antihypertensive effects of Nigella sativa oil in L-NAME-induced hypertensive rats. *Clinics*.

[58] Leong X. F., Rais Mustafa M., Jaarin K. (2013). *Nigella sativa* and its protective role in oxidative stress and hypertension. *Evidence-Based Complementary and Alternative Medicine*.

[59] Perveen T., Haider S., Zuberi N. A., Saleem S., Sadaf S., Batool Z. (2014). Increased 5-HT levels following repeated administration of Nigella sativa L. (black seed) oil produce antidepressant effects in rats. *Scientia Pharmaceutica*.

[60] Wilson M. A., Grillo C. A., Fadel J. R., Reagan L. P. (2015). Stress as a one-armed bandit: Differential effects of stress paradigms on the morphology, neurochemistry and behavior in the rodent amygdala. *Neurobiology of Stress*.

[61] Abulfadl Y. S., El-Maraghy N. N., Ahmed A. A. E., Nofal S., Badary O. A. (2018). Protective effects of thymoquinone on D-galactose and aluminum chloride induced neurotoxicity in rats: biochemical, histological and behavioral changes. *Neurological Research*.

[62] Hosseini M., Mohammadpour T., Karami R., Rajaei Z., Sadeghnia H. R., Soukhtanloo M. (2015). Effects of the hydro-alcoholic extract of *Nigella Sativa* on scopolamine-induced spatial memory impairment in rats and its possible mechanism. *Chinese Journal of Integrative Medicine*.

[63] Sahak M. K. A., Kabir N., Abbas G., Draman S., Hashim N. H., Hasan Adli D. S. (2016). The Role of* Nigella sativa* and its active constituents in learning and memory. *Evidence-Based Complementary and Alternative Medicine*.

[64] Sharaf R., Elsayed M. N., Mahran L. (2014). Neuroprotective effect of thymoquinone against lipopolysaccharide-induced Alzheimer’s disease in an animal model. *European Geriatric Medicine*.

[65] Sayeed M. S. B., Asaduzzaman M., Morshed H., Hossain M. M., Kadir M. F., Rahman M. R. (2013). The effect of *Nigella sativa* Linn. seed on memory, attention and cognition in healthy human volunteers. *Journal of Ethnopharmacology*.

[66] Alhebshi A. H., Gotoh M., Suzuki I. (2013). Thymoquinone protects cultured rat primary neurons against amyloid *β*-induced neurotoxicity. *Biochemical and Biophysical Research Communications*.

[67] Radad K., Moldzio R., Taha M., Rausch W.-D. (2009). Thymoquinone protects dopaminergic neurons against MPP+ and rotenone. *Phytotherapy Research*.

[68] Sedaghat R., Roghani M., Khalili M. (2014). Neuroprotective effect of thymoquinone, the nigella sativa bioactive compound, in 6-hydroxydopamine-induced hemi-parkinsonian rat model. *Iranian Journal of Pharmaceutical Research*.

[69] Alhebshi A. H., Odawara A., Gotoh M., Suzuki I. (2014). Thymoquinone protects cultured hippocampal and human induced pluripotent stem cells-derived neurons against *α*-synuclein-induced synapse damage. *Neuroscience Letters*.

[70] Norouzi F., Abareshi A., Anaeigoudari A. (2016). The effects of Nigella sativa on sickness behavior induced by lipopolysaccharide in male Wistar rats. *Avicenna Journal of Phytomedicine*.

[71] Bano F., Ahmed A., Parveen T., Haider S. (2014). Anxiolytic and hyperlocomotive effects of aqueous extract of Nigella sativa L. seeds in rats. *Pakistan Journal of Pharmaceutical Sciences*.

[72] Gilhotra N., Dhingra D. (2011). Thymoquinone produced antianxiety-like effects in mice through modulation of GABA and NO levels. *Pharmacological Reports*.

[73] Bin Sayeed M. S., Shams T., Hossain S. F. (2014). *Nigella sativa* L. seeds modulate mood, anxiety and cognition in healthy adolescent males. *Journal of Ethnopharmacology*.

[74] Seghatoleslam M., Alipour F., Shafieian R. (2016). The effects of Nigella sativa on neural damage after pentylenetetrazole induced seizures in rats. *Journal of Traditional and Complementary Medicine*.

[75] Abdollahi Fard M., Shojaii A. (2013). Efficacy of iranian traditional medicine in the treatment of epilepsy. *BioMed Research International*.

[76] Mostafa R., Moustafa Y., Mirghani Z. (2012). Thymoquinone alone or in combination with phenobarbital reduces the seizure score and the oxidative burden in pentylenetetrazole-kindled rats. *Oxidants and Antioxidants in Medical Science*.

[77] Akhondian J., Kianifar H., Raoofziaee M., Moayedpour A., Toosi M. B., Khajedaluee M. (2011). The effect of thymoquinone on intractable pediatric seizures (pilot study). *Epilepsy Research*.

[78] Abdel-Zaher A. O., Abdel-Rahman M. S., Elwasei F. M. (2010). Blockade of nitric oxide overproduction and oxidative stress by Nigella sativa oil attenuates morphine-induced tolerance and dependence in mice. *Neurochemical Research*.

[79] Sangi S., Ahmed S. P., Channa M. A., Ashfaq M., Mastoi S. M. (2008). A new and novel treatment of opioid dependence: Nigella sativa 500 mg. *Journal of Ayub Medical College*.

[80] Das B. K., Fatema U. K., Hossain M. S., Rahman R., Akbar M. A. (2014). Analgesic and anti-inflammatory activities of the fruit extract of *Ampelocissus latifolia*(Roxb) on laboratory animals. *British Journal of Pharmaceutical Research*.

[81] Pise H., Padwal S. (2017). Evaluation of anti-inflammatory activity of Nigella sativa: An experimental study. *National Journal of Physiology, Pharmacy and Pharmacology*.

[82] Zakaria A., Jais M. R., Ishak R. (2018). Analgesic properties of Nigella Sativa and Eucheuma Cottonii extracts. *Journal of Natural Science, Biology and Medicine*.

[83] Al-Ghamdi M. S. (2001). The anti-inflammatory, analgesic and antipyretic activity of *Nigella sativa*. *Journal of Ethnopharmacology*.

[84] Bashir M. U., Qureshi H. J. (2010). Analgesic effect of Nigella sativa seeds extract on experimentally induced pain in albino mice. *Journal of the College of Physicians and Surgeons Pakistan*.

[85] Rajsekhar S., Kuldeep B. (2011). Pharmacognosy and pharmacology of *Nigella sativa*. *International Research Journal of Pharmacy*.

[86] Houghton P. J., Zarka R., de las Heras B., Hoult J. R. S. (1995). Fixed oil of *Nigella sativa* and derived thymoquinone inhibit eicosanoid generation in leukocytes and membrane lipid peroxidation. *Planta Medica*.

[87] Mansour M., Tornhamre S. (2004). Inhibition of 5-lipoxygenase and leukotriene C4 synthase in human blood cells by thymoquinone. *Journal of Enzyme Inhibition and Medicinal Chemistry*.

[88] Boskabady M. H., Vahedi N., Amery S., Khakzad M. R. (2011). The effect of *Nigella sativa* alone, and in combination with dexamethasone, on tracheal muscle responsiveness and lung inflammation in sulfur mustard exposed guinea pigs. *Journal of Ethnopharmacology*.

[89] Boskabady M. H., Mohsenpoor N., Takaloo L. (2010). Antiasthmatic effect of Nigella sativa in airways of asthmatic patients. *Phytomedicine*.

[90] Ikhsan M., Hiedayati N., Maeyama K., Nurwidya F. (2018). Nigella sativa as an anti-inflammatory agent in asthma. *BMC Research Notes*.

[91] Saadat S., Mohammadi M., Fallahi M., keyhanmanesh R., Aslani M. R. (2015). The protective effect of *α*-hederin, the active constituent of Nigella sativa, on tracheal responsiveness and lung inflammation in ovalbumin-sensitized guinea pigs. *The Journal of Physiological Sciences*.

[92] Keyhanmanesh R., Bagban H., Nazemieh H., Bavil F. M., Alipour M. R. (2013). The main relaxant constituents of Nigella Sativa methanolic fraction on guinea pig tracheal chains. *Iranian Journal of Allergy, Asthma and Immunology*.

[93] Boskabady M. H., Keyhanmanesh R., Khamneh S., Ebrahimi M. A. (2011). The effect of Nigella sativa extract on tracheal responsiveness and lung inflammation in valbuminsensitized guinea pigs. *Clinics*.

[94] Salem A. M., Bamosa A. O., Qutub H. O. (2017). Effect of Nigella sativa supplementation on lung function and inflammatory mediators in partly controlled asthma: A randomized controlled trial. *Annals of Saudi Medicine*.

[95] Koshak A., Wei L., Koshak E. (2017). Nigella sativa supplementation improves asthma control and biomarkers: a randomized, double-blind, placebo-controlled trial. *Phytotherapy Research*.

[96] Boskabady M. H., Farhadi J. (2008). The possible prophylactic effect of Nigella sativa seed aqueous extract on respiratory symptoms and pulmonary function tests on chemical war victims: A randomized, double-blind, placebo-controlled trial. *The Journal of Alternative and Complementary Medicine*.

[97] Boskabady M. H., Javan H., Sajady M., Rakhshandeh H. (2008). The possible prophylactic effect of Nigella sativa seed extract in asthmatic patients. *Fundamental & Clinical Pharmacology*.

[98] Gheita T. A., Kenawy S. A. (2012). Effectiveness of Nigella sativa Oil in the management of rheumatoid arthritis patients: a placebo controlled study. *Phytotherapy Research*.

[99] Fathy M., Nikaido T. (2018). In vivo attenuation of angiogenesis in hepatocellular carcinoma by Nigella sativa. *Turkish Journal of Medical Sciences*.

[100] Yimer E. M., Surur A., Wondafrash D. Z., Gebre A. K. (2019). The effect of metformin in experimentally induced animal models of epileptic seizure. *Behavioural Neurology*.

[101] Lee C.-R., Cho I. H., Jeong B. C., Lee S. H. (2013). Strategies to minimize antibiotic resistance. *International Journal of Environmental Research and Public Health*.

[102] Ventola L. C. (2015). The antibiotic resistance crisis: causes and threats. *Pharm and Therap*.

[103] Andrade P. H. M., Schmidt Rondon E., Carollo C. A. (2015). Effect of Powdered Shells of the Snail *Megalobulimus lopesi* on Secondary-Intention Wound Healing in an Animal Model. *Evidence-Based Complementary and Alternative Medicine*.

[104] Theuretzbacher U. (2012). Accelerating resistance, inadequate antibacterial drug pipelines and international responses. *International Journal of Antimicrobial Agents*.

[105] Abdallah E. M. (2017). Black Seed (*Nigella sativa*) as antimicrobial drug: a mini-review. *Novel Approches in Drug Designing and Develop*.

[106] Hasan N. A., Nawahwi M. Z., Malek H. A. (2013). Antimicrobial activity of nigella sativa seed extract. *Sains Malaysiana*.

[107] Maryam A.-J., Fatimah A.-A., Ebtesam A.-K., Abdulrahman A.-S., Ineta B.-EL. (2016). In-vitro studies on the effect of *Nigella sativa* Linn., seed oil extract on Multidrug Resistant Gram positive and Gram negative bacteria. *Journal of Medicinal Plants*.

[108] Chaieb K., Kouidhi B., Jrah H., Mahdouani K., Bakhrouf A. (2011). Antibacterial activity of Thymoquinone, an active principle of *Nigella sativa* and its potency to prevent bacterial biofilm formation. *BMC Complementary and Alternative Medicine*.

[109] Salem E. M., Yar T., Bamosa A. O. (2010). Comparative study of Nigella Sativa and triple therapy in eradication of Helicobacter Pylori in patients with non-ulcer dyspepsia. *Saudi Journal of Gastroenterology*.

[110] Shokri H. (2016). A review on the inhibitory potential of Nigella sativa against pathogenic and toxigenic fungi. *Avicenna Journal of Phytomedicine*.

[111] Mahmoudvand H., Sepahvand A., Jahanbakhsh S., Ezatpour B., Mousavi S. A. A. (2014). Evaluation of antifungal activities of the essential oil and various extracts of *Nigella sativa* and its main component, thymoquinone against pathogenic dermatophyte strains. *Journal of Medical Mycology*.

[112] Aljabre S. H., Alakloby O. M., Randhawa M. A. (2015). Dermatological effects of *Nigella sativa*. *Journal of Dermatology & Dermatologic Surgery*.

[113] Piras A., Rosa A., Marongiu B. (2013). Chemical composition and in vitro bioactivity of the volatile and fixed oils of *Nigella sativa* L. extracted by supercritical carbon dioxide. *Industrial Crops and Products*.

[114] Taha M., Azeiz A., Saudi W. (2010). Antifungal effect of thymol, thymoquinone and thymohydroquinone against yeasts, dermatophytes and non-dermatophyte molds isolated from skin and nails fungal infections. *Egyptian Journal of Biochemistry and Molecular Biology*.

[115] Forouzanfar F., Fazly Bazzaz B. S., Hosseinzadeh H. (2014). Black cumin (Nigella sativa) and its constituent (thymoquinone): A review on antimicrobial effects. *Iranian Journal of Basic Medical Sciences*.

[116] Barakat E. M. F., El Wakeel L. M., Hagag R. S. (2013). Effects of Nigella sativa on outcome of hepatitis C in Egypt. *World Journal of Gastroenterology*.

[117] Onifade A., Jewell A., Adedeji W. (2013). *Nigella Sativa* Concoction induced sustained seroreversion in HIV patient. *African Journal of Traditional, Complementary and Alternative Medicines*.

[118] Onifade A. A., Jewell A. P., Okesina A. B. (2015). Seronegative conversion of an HIV positive subject treated with Nigella sativa and honey. *African Journal of Infectious Diseases*.

[119] Assi M. A., Noor M. H. M., Bachek N. F. (2016). The various effects of Nigella sativa on multiple body systems in human and animals. *Pertanika Journal of Scholarly Research Reviews*.

[120] Abd El-Hack M. E., Alagawany M., Farag M. R., Tiwari R., Karthik K., Dhama K. (2016). Nutritional, healthical and therapeutic efficacy of black cumin (Nigella sativa) in animals, poultry and humans. *International Journal of Pharmacology*.

[121] Bafghi A. F., Vahidi A. R., Anvari M. H., Barzegar K., Ghafourzadeh M. (2011). The in vivo antileishmanial activity of alcoholic extract from Nigella sativa seeds. *African Journal of Microbiology Research*.

[122] Okeola V. O., Adaramoye O. A., Nneji C. M., Falade C. O., Farombi E. O., Ademowo O. G. (2011). Antimalarial and antioxidant activities of methanolic extract of Nigella sativa seeds (black cumin) in mice infected with Plasmodium yoelli nigeriensis. *Parasitology Research*.

[123] Schneider-Stock R., Fakhoury I. H., Zaki A. M., El-Baba C. O., Gali-Muhtasib H. U. (2014). Thymoquinone: fifty years of success in the battle against cancer models. *Drug Discovery Therapy*.

[124] Arafa E.-S. A., Zhu Q., Shah Z. I. (2011). Thymoquinone up-regulates PTEN expression and induces apoptosis in doxorubicin-resistant human breast cancer cells. *Mutation Research - Fundamental and Molecular Mechanisms of Mutagenesis*.

[125] Periasamy V. S., Athinarayanan J., Alshatwi A. A. (2016). Anticancer activity of an ultrasonic nanoemulsion formulation of Nigella sativa L. essential oil on human breast cancer cells. *Ultrasonics Sonochemistry*.

[126] Ng W. K., Yazan L. S., Ismail M. (2011). Thymoquinone from *Nigella sativa* was more potent than cisplatin in eliminating of SiHa cells via apoptosis with down-regulation of Bcl-2 protein. *Toxicology in Vitro*.

[127] Effenberger K., Breyer S., Schobert R. (2010). Terpene conjugates of the Nigella sativa seed-oil constituent thymoquinone with enhanced efficacy in cancer cells. *Chemistry & Biodiversity*.

[128] Zhang M., Du H., Huang Z. (2018). Thymoquinone induces apoptosis in bladder cancer cell via endoplasmic reticulum stress-dependent mitochondrial pathway. *Chemico-Biological Interactions*.

[129] Zhang Y., Fan Y., Huang S. (2018). Thymoquinone inhibits the metastasis of renal cell cancer cells by inducing autophagy via AMPK/mTOR signaling pathway. *Cancer Science*.

[130] Kou B., Kou Q., Ma B. (2018). Thymoquinone inhibits metastatic phenotype and epithelial-mesenchymal transition in renal cell carcinoma by regulating the LKB1/AMPK signaling pathway. *Oncology Reports*.

[131] Kou B., Liu W., Zhao W. (2017). Thymoquinone inhibits epithelial-mesenchymal transition in prostate cancer cells by negatively regulating the TGF-*β*/Smad2/3 signaling pathway. *Oncology Reports*.

[132] Elkhoely A., Hafez H. F., Ashmawy A. M. (2015). Chemopreventive and therapeutic potentials of thymoquinone in HepG2 cells: Mechanistic perspectives. *Journal of Natural Medicines*.

[133] Shahin Y. R., Elguindy N. M., Abdel Bary A., Balbaa M. (2018). The protective mechanism of *Nigella sativa* against diethylnitrosamine-induced hepatocellular carcinoma through its antioxidant effect and EGFR/ERK1/2 signaling. *Environmental Toxicology*.

[134] Kabil N., Bayraktar R., Kahraman N. (2018). Thymoquinone inhibits cell proliferation, migration, and invasion by regulating the elongation factor 2 kinase (eEF-2K) signaling axis in triple-negative breast cancer. *Breast Cancer Research and Treatment*.

[135] Salim E. I., Fukushima S. (2003). Chemopreventive potential of volatile oil from black cumin (Nigella sativa L.) seeds against rat colon carcinogenesis. *Nutrition and Cancer*.

[136] Gali-Muhtasib H., Ocker M., Kuester D. (2008). Thymoquinone reduces mouse colon tumor cell invasion and inhibits tumor growth in murine colon cancer models. *Journal of Cellular and Molecular Medicine*.

[137] Salim E. I. (2010). Cancer chemopreventive potential of volatile oil from black cumin seeds, Nigella sativa L., in a rat multi-organ carcinogenesis bioassay. *Oncology Letters*.

[138] Alenzi F. Q., El-Sayed El-Bolkiny Y., Salem M. L. (2010). Protective effects of Nigella sativa oil and thymoquinone against toxicity induced by the anticancer drug cyclophosphamide. *British Journal of Biomedical Science*.

[139] Mu G.-G., Zhang L.-L., Li H.-Y., Liao Y., Yu H.-G. (2015). Thymoquinone pretreatment overcomes the insensitivity and potentiates the antitumor effect of gemcitabine through abrogation of notch1, PI3K/Akt/mTOR regulated signaling pathways in pancreatic cancer. *Digestive Diseases and Sciences*.

[140] Şakalar Ç., İzgi K., İskender B. (2016). The combination of thymoquinone and paclitaxel shows anti-tumor activity through the interplay with apoptosis network in triple-negative breast cancer. *Tumor Biology*.

[141] Khalife R., Hodroj M. H., Fakhoury R., Rizk S. (2016). Thymoquinone from nigella sativa seeds promotes the antitumor activity of noncytotoxic doses of topotecan in human colorectal cancer cells in vitro. *Planta Medica*.

[142] Gurunath S., Pandian Z., Anderson R. A., Bhattacharya S. (2011). Defining infertility-a systematic review of prevalence studies. *Human Reproduction Update*.

[143] Aitken R. J., Smith T. B., Jobling M. S., Baker M. A., De Iuliis G. N. (2014). Oxidative stress and male reproductive health. *Asian Journal of Andrology*.

[144] Wright C., Milne S., Leeson H. (2014). Sperm DNA damage caused by oxidative stress: modifiable clinical, lifestyle and nutritional factors in male infertility. *Reproductive BioMedicine Online*.

[145] Awah F. M., Uzoegwu P. N., Ifeonu P. (2012). Free radical scavenging activity, phenolic contents and cytotoxicity of selected Nigerian medicinal plants. *Food Chemistry*.

[146] Ashraf S. S., Rao M. V., Kaneez F. S. (2011). Nigella sativa extract as a potent antioxidant for petrochemical-induced oxidative stress. *Journal of Chromatographic Science (JCS)*.

[147] Parandin R., Yousofvand N., Ghorbani R. (2012). The enhancing effects of alcoholic extract of Nigella sativa seed on fertility potential, plasma gonadotropins and testosterone in male rats. *Iranian Journal of Reproductive Medicine*.

[148] Kolahdooz M., Nasri S., Modarres S. Z., Kianbakht S., Huseini H. F. (2014). Effects of Nigella sativa L. seed oil on abnormal semen quality in infertile men: A randomized, double-blind, placebo-controlled clinical trial. *Phytomedicine*.

[149] Badary O. A., Al-Shabanah O. A., Nagi M. N., Al-Bekairi A. M., Elmazar M. M. A. (1998). Acute and subchronic toxicity of thymoquinone in mice. *Drug Development Research*.

[150] Bensiameur-Touati K., Kacimi G. I., Haffaf E.-M., Berdja S., Aouichat-Bouguerra S. (2017). In vivo subacute toxicity and antidiabetic effect of aqueous extract of* Nigella sativa*. *Evidence-Based Complementary and Alternative Medicine*.

[151] Zaoui A., Cherrah Y., Mahassini N., Alaoui K., Amarouch H., Hassar M. (2002). Acute and chronic toxicity of *Nigella sativa* fixed oil. *Phytomedicine*.

